# Polymerization Kinetics and Development of Polymerization Shrinkage Stress in Rapid High-Intensity Light-Curing

**DOI:** 10.3390/polym14163296

**Published:** 2022-08-12

**Authors:** Matej Par, Phoebe Burrer, Katica Prskalo, Saskia Schmid, Anna-Lena Schubiger, Danijela Marovic, Zrinka Tarle, Thomas Attin, Tobias T. Tauböck

**Affiliations:** 1 Department of Endodontics and Restorative Dentistry, School of Dental Medicine, University of Zagreb, Gunduliceva 5, 10000 Zagreb, Croatia; 2 Department of Conservative and Preventive Dentistry, Center of Dental Medicine, University of Zurich, Plattenstrasse 11, 8032 Zurich, Switzerland

**Keywords:** resin composites, light-curing, bulk-fill composites, rapid light-curing

## Abstract

This study investigated polymerization kinetics, linear shrinkage, and shrinkage stress development for six contemporary composite materials of different viscosities cured using radiant exitances of 1100–2850 mW/cm^2^. Real-time measurements of degree of conversion, linear shrinkage, and shrinkage stress were performed over 5 min using Fourier-transform infrared spectrometry, a custom-made linometer, and a custom-made stress analyzer, respectively. For most tested variables, the factor “material” had a higher effect size than the factor “curing protocol”. Maximum polymerization rate and maximum shrinkage stress rate were the most affected by changes in curing conditions. In contrast, no significant effects of curing conditions were identified within each material for shrinkage stress values measured at the end of the 5 min observation period. Linear shrinkage and shrinkage stress values measured after 5 min were closely correlated (R = 0.905–0.982). The analysis of polymerization kinetics suggested that the two composites specifically designed for rapid light-curing responded to higher radiant exitances differently than other composites. Polymerization kinetics and shrinkage stress behavior of contemporary restorative composite materials of different viscosities were overall more affected by material type than differences in curing conditions. Subtle differences in polymerization kinetics behavior shown by the two composites specifically designed for rapid high-intensity light-curing did not translate into significant differences in the development of polymerization shrinkage stress.

## 1. Introduction

Motivated by the desire to shorten chair-time and reduce technique sensitivity, the development of resin composites and application techniques aims for procedural simplification. A major simplification of restorative treatment is made possible by using bulk-fill composites which have, at least considering the available evidence, successfully fulfilled the expectations and provide properties comparable to those of conventional composites [1]. Another recent development of restorative procedures that is aligned with the trend for shortening clinical application includes resin composites specifically intended for ultra-fast (3 s) light-curing and the accompanying curing unit capable of producing the required high radiant exitance of 3000 mW/cm^2^ [2,3,4,5].

Although the attempts to light-cure resin composites with high intensity (over 1000 mW/cm^2^) originated more than two decades ago, it was not until the recent development of lightweight, battery-powered LED devices and compositional modifications of resin composites that such curing became clinically feasible [6]. One sculptable (PowerFill) and one flowable composite (PowerFlow, both by Ivoclar Vivadent) have been developed and advertised specifically for rapid high-intensity light-curing [2,3,4,5]. These materials feature two main compositional modifications: a Norrish type I photoinitiator (both PowerFill and PowerFlow) and a beta-allyl sulfone addition-fragmentation chain transfer (AFCT) reagent (only in PowerFill). These modifications allow the polymer network to develop more homogeneously and attain better mechanical properties [7,8,9]. Before these composites were launched, another brand of bulk-fill composite (Filtek One by 3M corporation) had already incorporated an AFCT agent but has not been advertised as being specifically designed for high-intensity curing. In that material, the AFCT agent is reportedly included to reduce shrinkage stress [10].

The attractiveness of composites designed for rapid high-intensity light-curing has motivated intensive research of their properties, which is still mostly limited to in vitro studies. These materials have been shown to reach degree of conversion values comparable to conventional composites, regardless of being subjected to a conventional or high-intensity light-curing [3]. A study on PowerFill reported comparable curing kinetics, depth of cure, flexural strength/modulus, and indentation hardness/modulus as its predecessor that polymerizes via a conventional free-radical polymerization [8]. High-intensity light-curing of PowerFill also demonstrated similar viscoelastic properties and cell toxicity as conventional curing [11]. In a study on shrinkage and shrinkage stress of PowerFill and PowerFlow, a reduction in shrinkage strain accompanied by an increase in shrinkage stress was attributed to reduced network mobility due to high-intensity curing [2]. Comparable flexural strength and modulus, as well as depth-sensing indentation properties, were reported for PowerFill cured with high-intensity and conventional curing protocols, 24 h post-cure and after artificial aging by thermocycling [9]. The risk of high-intensity light-curing diminishing the degree of conversion of the flowable composite PowerFlow compared to conventional curing has been reported in two studies [4,12]; however, this is with the benefit of improved crosslinking density [4]. In contrast, a possible decline in crosslinking density has been reported for the high-viscosity counterpart PowerFill [9]. There is also evidence of a negative effect of high-intensity curing on flexural modulus of PowerFill [5]; however, the clinical relevance of such an effect remains unclear. Another indication of a potentially inferior material performance was reported in a marginal integrity study in which a high-intensity curing protocol produced an initially comparable marginal integrity to conventional curing, whereas artificial aging by means of thermo-mechanical loading revealed significantly inferior marginal integrity of flowable composites (both for PowerFlow and a conventional flowable reference bulk-fill material) cured with the high-intensity protocol [13]. Additionally, high-viscosity materials with AFCT-modified polymerization (PowerFill and Filtek One) showed better resistance to artificial aging and better maintained their mechanical properties compared to the flowable material PowerFlow [14]. Garoushi et al. [15] recommended that high-intensity curing should be used only with the composites specifically designed for this protocol, as using it with other conventional composites can diminish the degree of conversion and lead to inferior mechanical properties. However, this recommendation was based on the comparison of PowerFill with only one conventional composite.

As “high-intensity” LED curing units with radiant exitances between 1000 and 3000 mW/cm^2^ are becoming increasingly common on the dental market [16], possible consequences of fast polymerization on the polymerization shrinkage behavior of restorative composites (both conventional and those specifically designed for high-intensity light-curing) should be addressed. Volumetric shrinkage, which unavoidably occurs in resin composites due to the shortening of intermolecular distances during polymerization, leads to the formation of stresses at the bonded tooth/restoration interface and causes local bond failure, which is related to multiple undesired outcomes, including marginal discoloration, postoperative sensitivity, and secondary caries. Hence, the present study investigated polymerization kinetics, linear shrinkage, and shrinkage stress development for six contemporary composite materials characterized by different viscosities (flowable, sculptable, and packable). The hypotheses were that (I) polymerization kinetics and shrinkage behavior would be affected by radiant exposures in the range of 1100–2850 mW/cm^2^, and that (II) different materials would exhibit different polymerization kinetics and shrinkage behavior.

## 2. Materials and Methods

### 2.1. Composite Materials and Curing Protocols

The manufacturer’s information about six investigated composite materials is shown in Table 1.

Composite specimens were light-cured using an LED curing unit (Bluephase PowerCure; Ivoclar Vivadent, Schaan, Liechtenstein) with a light guide tip diameter of 9 mm, and an emission wavelength range of 390–500 nm. Radiant exitance of the curing unit was measured and periodically checked using a calibrated and NIST-referenced UV-vis spectrophotometer system (MARC; BlueLight Analytics, Halifax, Canada). The following three curing protocols pre-set by the curing unit manufacturer were used:

- “3-s” protocol: 3 s @ 2850 mW/cm^2^ (8.55 J/cm^2^);
- “5-s” protocol: 5 s @ 2050 mW/cm^2^ (10.25 J/cm^2^);
- “10-s” protocol: 10 s @ 1100 mW/cm^2^ (11.00 J/cm^2^).

### 2.2. Degree of Conversion

Degree of conversion was measured in real time using a Fourier-transform infrared (FTIR) spectrometer in the attenuated total reflectance mode (Nicolet iS50, Thermo Fisher, Madison, WI, USA). Composite specimens contained within cylindrical Teflon molds (diameter = 6 mm, thickness = 1.5 mm) were placed on top of a diamond crystal. Mold openings were covered with a polyethylene terephthalate (PET) film and the light-curing unit (Bluephase PowerCure) was centered at a distance of 0.1 mm above the specimen surface. FTIR spectra were collected successively over 5 min after the light-curing unit has been activated, at a rate of 2 s^−1^, with 4 scans per spectrum and a spectral resolution of 8 cm^−1^. Six experimental runs per group were performed (*n* = 6).

Real-time degree of conversion (*DC*) was calculated from the absorbance ratio of the spectral band at 1638 cm^−1^ (aliphatic C=C) and a *reference* spectral band according to the following expression:

$$DC\;\left(\%\right)=\left[1-\frac{{\left(1638\;{\mathrm{cm}}^{-1}/reference\right)}_{peak\;height\;after\;curing}\;}{{\left(1638\;{\mathrm{cm}}^{-1}/reference\right)}_{peak\;height\;before\;curing}}\right]\times 100$$

The spectral band at 1608 cm^−1^ (aromatic C⋯C) was used as a *reference* for all composites except for Filtek One, for which, due to the lack of the C⋯C band, an alternative band at 1600 cm^−1^ (C-H stretching) was used as a *reference* [17].

The final *DC* values measured at the end of the 5 min observation period were recorded as DC_5min_. After plotting the real-time *DC* data as a function of time, first derivatives were calculated as a measure of the polymerization rate. The polymerization rate was plotted against time to determine the maximum reaction rate (R_DC_max) and time to reach maximum reaction rate (t_DC_max) [18].

As an additional evaluation of polymerization kinetics, the DC vs. time curves were fitted by a four-parameter exponential sum function y = a ∗ (1 − e^−bx^) + c ∗ (1 − e^−dx^) [19]. This approach has been frequently used for evaluating polymerization kinetics during the gel phase (parameters a and b) and the glass phase (parameters c and d) [18,19,20,21,22,23].

### 2.3. Linear Shrinkage

Linear shrinkage was investigated using a custom-made linometer according to previous studies [24,25,26,27,28]. On a solid metal frame of the linometer, a thin aluminum platelet (12 × 12 mm, thickness = 0.25 mm, m = 0.4 g) was loosely placed. From the bottom side of the platelet, a perpendicular diaphragm extended into a recess of the infrared measuring sensor. On the upper surface of the aluminum platelet, a cylindrical composite specimen of standardized volume (V = 42 mm^3^, d = 6 mm) was placed and flattened to a thickness of 1.5 mm using a glass plate. The light guide tip of the curing unit was placed directly above the glass plate. Change in the composite specimen volume due to polymerization shrinkage displaced the diaphragm in the vertical direction, which was recorded by the infrared sensor. Real-time data were recorded at 5 s^−1^ for 5 min after the start of light-curing, with an accuracy of 0.1 µm. To simulate intraoral temperature after rubber-dam placement, the experiment was performed in a temperature-controlled chamber at 25 ± 1 °C [27]. Six experimental runs were performed for each group (*n* = 6). The final linear shrinkage values measured at the end of the 5 min observation period were recorded as LS_5min_.

### 2.4. Shrinkage Stress

Shrinkage stress was measured using a custom-made stress analyzer according to previous studies [24,25,26,27,29]. The testing device was semi-rigid (compliance = 0.4 μm/N) in order to simulate the partial stress relief by elastic displacement of dental hard tissues [27]. The same standardized composite amount as used for linear shrinkage measurements (V = 42 mm^3^, d = 6 mm) was placed on the flat surface of a metal cylinder connected to a load cell (PM 11-K; Mettler, Greifensee, Switzerland). The composite was pressed using a sandblasted/silanized glass plate to obtain a thickness of 1.5 mm, which resulted in a cylindrical specimen with a base surface area of 28 mm^2^. As the bases of composite specimens were bonded to the metal cylinder and the glass plate, the ratio of bonded to unbonded surface area (C-factor) amounted to 2.0. Bonding of the composite to the glass plate and metal cylinder was ensured by sandblasting their surfaces with aluminum oxide (50 μm; Renfert, Hilzingen, Germany), rinsing with a stream of demineralized water for 30 s, drying using a pressurized air stream, and treating with a silane-containing primer, Monobond Plus (Ivoclar Vivadent). Visual inspection with a stereomicroscope at 40× magnification (M3Z; Leica/Wild, Heerbrugg, Switzerland) was used to confirm that no aluminum oxide particles remained on the sandblasted surfaces. Immediately after securing the composite specimen within the measuring device, light-curing was initiated through the glass plate as described above for the linear shrinkage measurements. The real-time development of force due to polymerization shrinkage was recorded by the load cell at 5 s^−1^ and an accuracy of 0.001 N. The data were collected for 5 min after the start of light-curing, inside a temperature-controlled chamber at 25 ± 1 °C. Six experimental runs were performed for each group (*n* = 6). Shrinkage stress was calculated by dividing the obtained shrinkage force values by the surface area of the composite specimen base (28 mm^2^). The final shrinkage stress values measured at the end of the 5 min observation period were recorded as SS_5min_. The curves of polymerization shrinkage stress vs. time were plotted and the first derivatives of these curves were used to represent the shrinkage stress rate. From the plots of first derivatives vs. time, the following parameters of shrinkage kinetics were estimated: maximum shrinkage stress rate (R_SS_max) and time to achieve maximum shrinkage stress rate (t_SS_max).

### 2.5. Statistical Analysis

For each of the tested properties (degree of conversion, linear shrinkage, and shrinkage stress), the total number of specimens was 108 (6 materials × 3 curing protocols × 6 experimental runs). Normality of distribution was verified using the Shapiro–Wilk test and normal Q-Q diagrams. The data were analyzed using two-way ANOVA with the factors “material” and “curing protocol”. Since there were statistically significant interactions between these two factors, separate one-way ANOVAs were performed for each factor at a fixed level of the other factor. Tukey’s post-hoc adjustment was used for multiple comparisons and statistically similar values at the significance level of α = 0.05 were marked as statistically homogeneous groups. Partial eta-squared statistics were used to estimate the effect size for the factors “material” and “curing protocol”. Pearson correlation analysis was used to evaluate the relationship between linear shrinkage and shrinkage stress. A principal component analysis with varimax rotation was used to explore the amount of covariance among the tested variables. For the fit parameters of the double exponential sum function used for describing polymerization kinetics, the 95% confidence intervals were calculated as a measure of uncertainty. The statistical analysis was performed using SPSS (version 25; IBM, Armonk, NY, USA) at an overall α = 0.05.

## 3. Results

Table 2 shows the results of the two-way ANOVA for the factors “material” and “curing protocol”. Statistically significant effects of these factors were identified for all variables, with the exception of the curing protocol showing no significant effect on SS_5min_. Partial eta-squared values indicate that the factor “material” had a higher effect size than the factor “curing protocol” for all investigated variables, except for t_SS_max.

**Figure 1:**
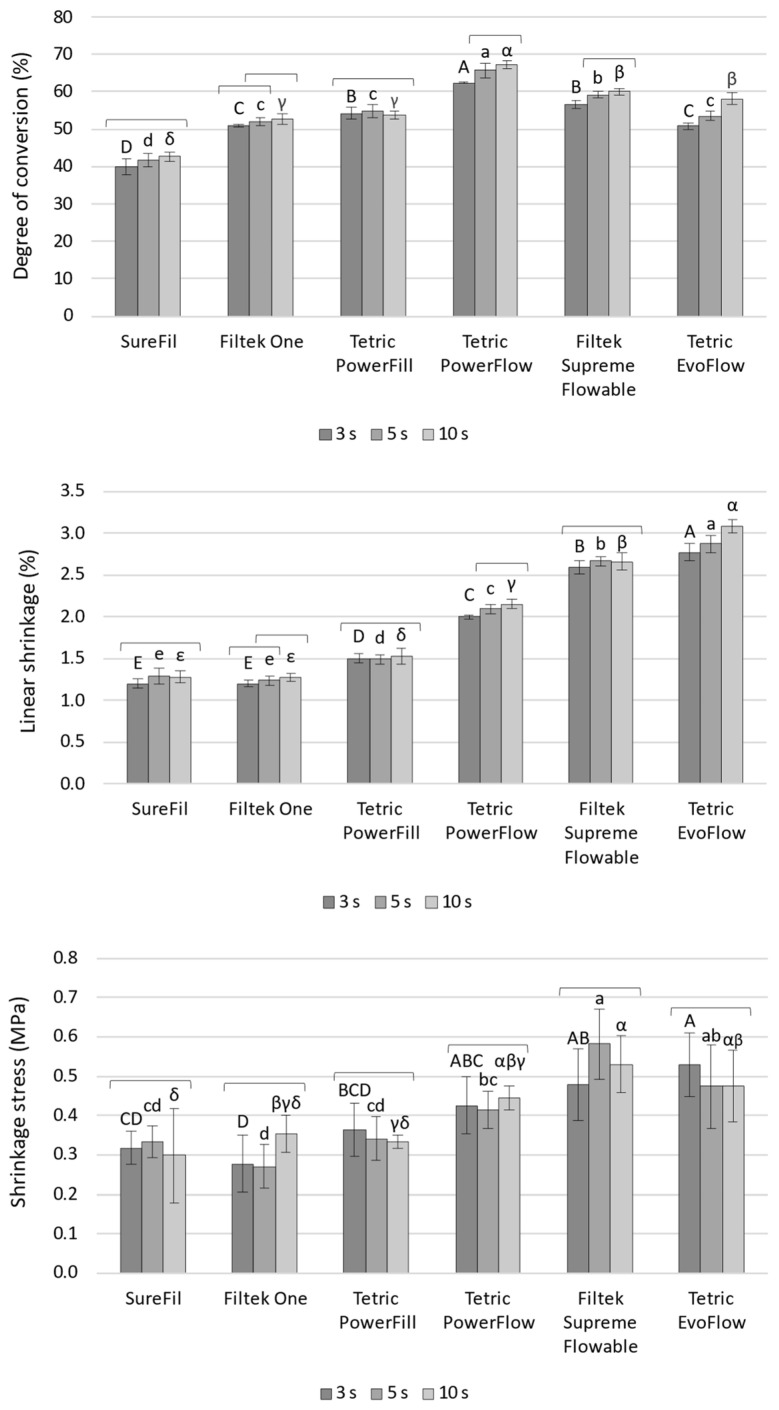
Degree of conversion, linear shrinkage, and shrinkage stress measured at the end of the 5 min observation period (mean values ± 1 SD). Statistically similar values among curing protocols are connected with square brackets. Statistically similar values among materials are denoted by same uppercase, lowercase, and Greek letters for the 3, 5, and 10 s curing protocols, respectively.

The static parameters measured at the end of the 5 min observation period (DC_5min_, LS_5min_, and SS_5min_) are shown in Figure 1. For SureFil and Tetric PowerFill, curing protocols had no significant effect on DC_5min_ and LS_5min_. In contrast, for Filtek One, Tetric PowerFlow, and Tetric EvoFlow, a statistically significant effect of curing protocols was observed for DC_5min_ and LS_5min_, with the overall ranking of 10 s > 5 s > 3 s (with some statistically similar overlapping observed for individual materials). The analogous effect of curing protocols on DC_5min_ was observed for Filtek Supreme Flowable; however, this was without the effect on LS_5min_. The parameter SS_5min_ behaved differently than DC_5min_ and LS_5min_ by showing no significant effect of curing protocols for any of the tested materials.

Comparisons of LS_5min_ values among the materials showed the following ranking: SureFil = Filtek One ≤ Tetric PowerFill ≤ Tetric PowerFlow ≤ Filtek Supreme Flowable ≤ Tetric EvoFlow. This ranking follows the composite’s filler load, as the materials in Figure 1 are presented in descending order of their filler load. For SS_5min_, the same material ranking follows that mentioned above for LS_5min_, although with more overlapping of statistically similar values among the materials. The more pronounced overlap was caused by higher data variability for SS_5min_ compared to LS_5min_.

**Figure 2:**
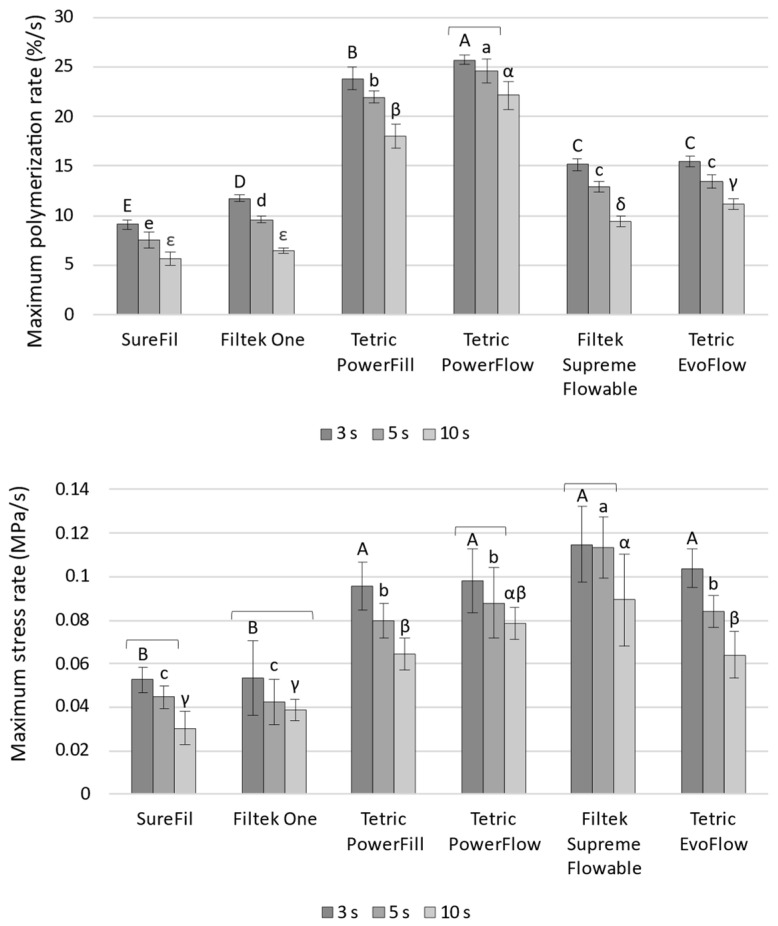
Maximum polymerization rate and maximum shrinkage stress rate (mean values ± 1 SD). Statistically similar values among curing protocols are connected with square brackets. Statistically similar values among materials are denoted by same uppercase, lowercase, and Greek letters for the 3, 5, and 10 s curing protocols, respectively.

Figure 2 shows the kinetic parameters R_DC_max and R_SS_max. Among all the parameters in this study, R_DC_max discriminated best among the curing protocols, providing the highest frequency of statistically significant comparisons with the ranking of curing protocols of 3 s > 5 s > 10 s. The only case in which this statistically significant effect was not identified was the comparison of 3 s vs. 5 s for Tetric PowerFlow. A similar general pattern of 3 s > 5 s > 10 s was observed for R_SS_max, although with more frequent overlapping of statistically similar values. In inter-material comparisons, Tetric PowerFlow and Tetric PowerFill stood out from the rest of the materials by showing the highest R_DC_max. However, this distinction was much less apparent for R_SS_max, as Tetric PowerFlow and Tetric PowerFill showed statistically similar R_SS_max values as Filtek Supreme Flowable and Tetric EvoFlow.

**Figure 3:**
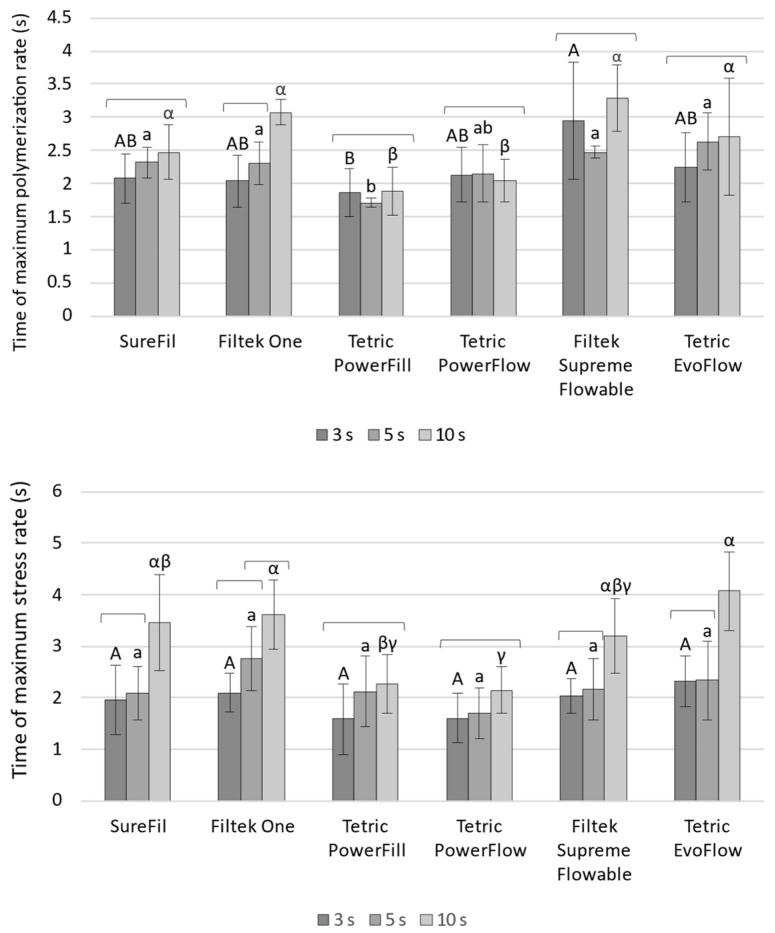
Time of maximum polymerization rate and time of maximum shrinkage stress rate (mean values ± 1 SD). Statistically similar values among curing protocols are connected with square brackets. Statistically similar values among materials are denoted by same uppercase, lowercase, and Greek letters for the 3, 5, and 10 s curing protocols, respectively.

Figure 3 shows the kinetic parameters t_DC_max and t_SS_max. These parameters discriminated poorly among the curing protocols; a statistically significant effect of curing protocols on t_DC_max was identified only for Filtek One cured with the 10 s protocol, whereas t_SS_max values showed a statistically significant effect only for the 10 s protocol for the following materials: SureFil, Filtek One, Filtek Supreme Flowable, and Tetric EvoFlow. The inter-material comparisons for t_DC_max and t_SS_max also showed poor discriminative capability, with most comparisons showing statistically non-significant results.

**Figure 4:**
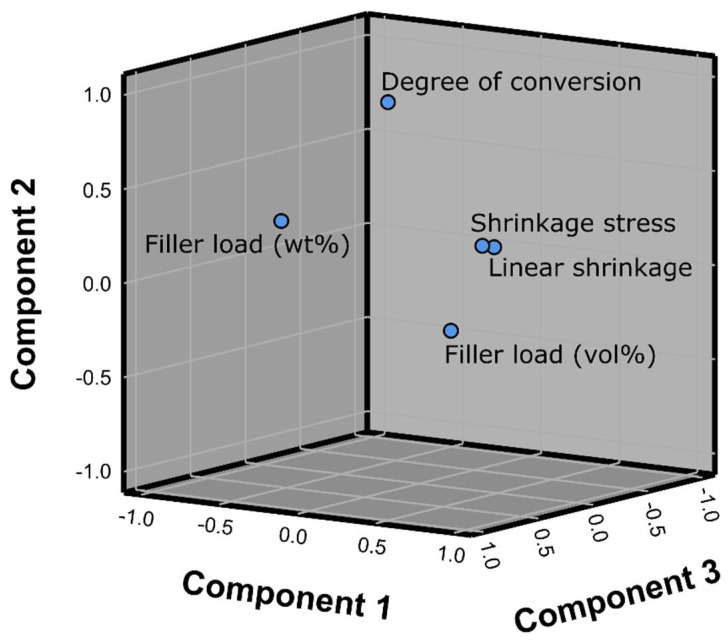
The component loading plot in rotated space for representing the amount of covariance among degree of conversion, linear shrinkage after 5 min, shrinkage stress after 5 min, and filler load expressed in wt% and vol%.

**Figure 5:**
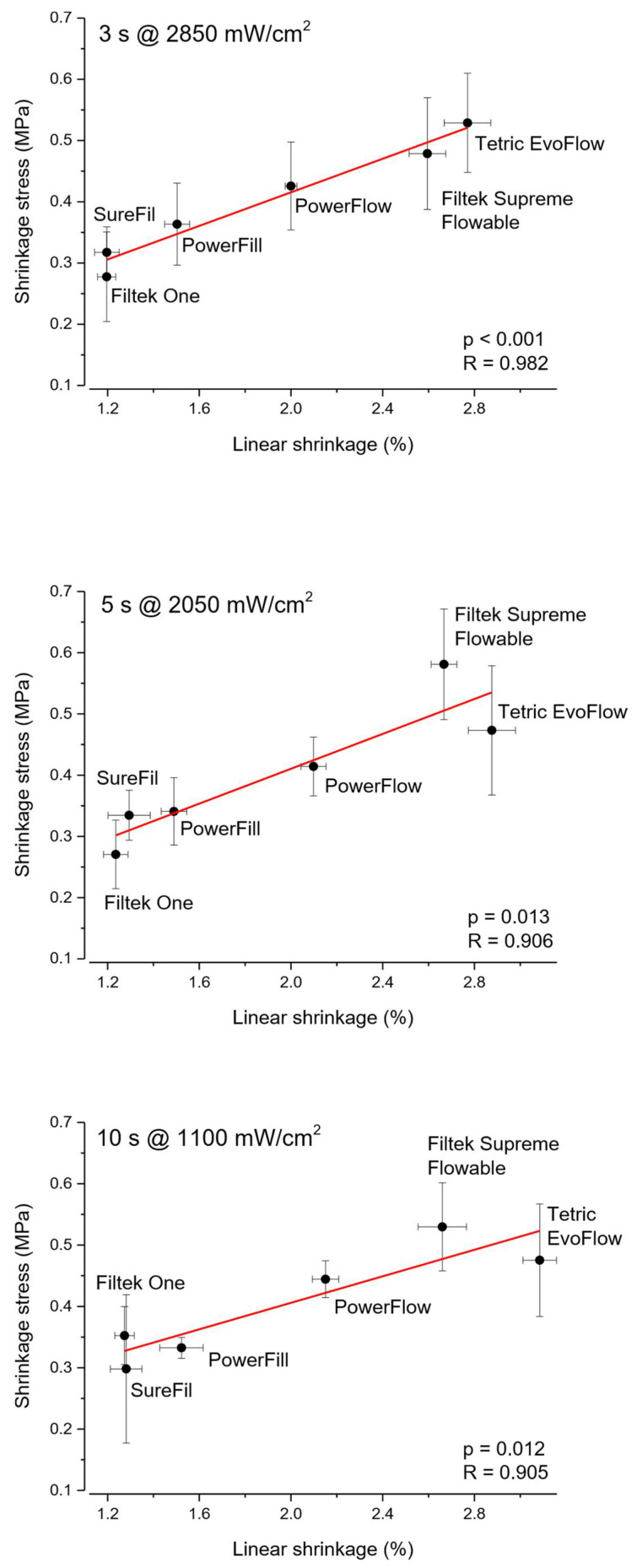
Scatterplots of shrinkage stress as a function of linear shrinkage and results of Pearson correlation analysis. Error bars represent ± 1 standard deviation.

Figure 4 shows the loading plot of the first three principal components in rotated space, which is useful for visualizing relative amounts of covariance among static parameters (DC_5min_, LS_5min_, and SS_5min_) and filler load expressed in wt% and vol%. The spatial relationships among variables in the rotated space indicate relative amounts of their joint variability. The relative closeness of linear shrinkage and shrinkage stress indicates that these variables share a high amount of covariance, while also being closer to filler (vol%) than to any other variables. The high amount of joint variability for linear shrinkage and shrinkage stress is also demonstrated by Pearson correlation analysis and the associated plots shown in Figure 5. Pearson’s R amounted to 0.905–0.982, indicating that linear shrinkage and shrinkage stress were very closely correlated.

**Figure 6:**
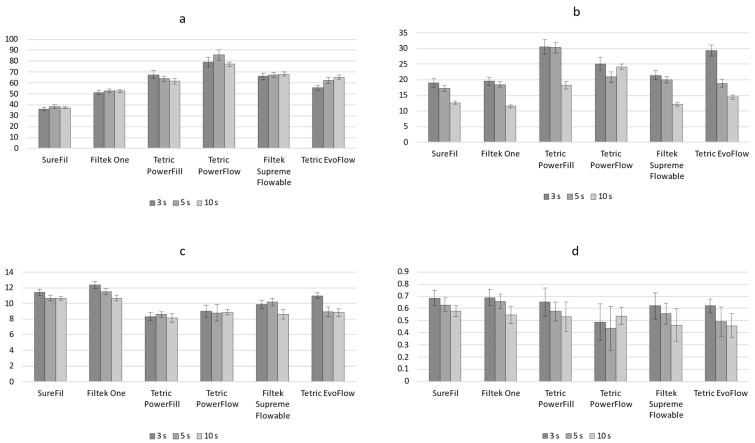
Fit parameters (**a**–**d**) of double exponential function used for describing polymerization kinetics. Error bars represent 95% confidence intervals.

Figure 6 shows the fit parameters of the double exponential function used for describing polymerization kinetics. All parameters were affected by the curing protocols, while the largest extent of change was identified in the parameter “b”, which was 33.5–50.7% lower in the 10 s protocol compared to the 3 s protocol. An exception was identified for PowerFlow, which showed much lower variations in the parameter “b” due to different curing protocols (3.9–13.4%). The parameter “a” was reduced with increasing radiant exitance, except for PowerFill and PowerFlow, which showed different patterns; in PowerFill, an increase due to higher radiant exitance was observed, whereas in PowerFlow no clear dependence on radiant exitance was discernible. An inverse behavior to that of the parameter “a” was observed for the parameter “c”, which increased with increasing radiant exitance in all materials, except in PowerFill and PowerFlow. The latter materials showed no change in parameter “c” due to different curing protocols. The parameter “d” shows an increasing pattern as radiant exitance increases, except for PowerFlow, which showed no differences among curing protocols.

## 4. Discussion

This study investigated how light-curing using 1100–2850 mW/cm^2^ affected the development of polymerization kinetics and shrinkage stress of flowable, sculptable, and packable resin composites, including two bulk-fill composites specifically designed for rapid high-intensity light-curing. The relationship between polymerization and shrinkage kinetics was explored using different curing protocols and real-time measurements of degree of conversion, linear shrinkage, and shrinkage stress. Different combinations of radiant exposure/curing time affected the measured properties to different extents, with maximum polymerization rate and maximum shrinkage stress rate being the most sensitive to changes in curing conditions. The analysis of polymerization kinetics also suggested that the composites specifically designed for rapid light-curing responded to higher radiant exitances differently than other composites. As polymerization kinetics and shrinkage behavior were significantly affected by curing conditions and material type, both hypotheses were accepted.

Curing was performed using three preset modes of a contemporary wide-spectrum LED device, with radiant exposures ranging between 8.55 and 11.00 J/cm^2^. This differed from an idealized approach of keeping the radiant exposure constant for all curing protocols and represents an unavoidable result of using an unmodified commercial device with preset parameters of curing protocols. An additional source of variation was the deviation from nominal curing unit specifications, which are commonly tolerated by manufacturers. For comparison, in a previous study, another curing unit of the same type was used that produced 20% higher radiant exitance values for the “3-s” curing protocol (3440 mW/cm^2^) compared to 2850 mW/cm^2^ in the present study [25]. These variations are considerable and remain uncontrollable in a clinical setting, where the practitioner cannot precisely evaluate the radiant exitance of a particular curing unit due to the lack of a laboratory-grade UV-vis spectrometer. Instead, practitioners follow manufacturer instructions, which for the curing unit in the present study specify the following radiant exitances (mW/cm^2^): 1200, 2000, and 3000, which amounts to 92%, 103%, and 95% of the actual values measured for the 10, 5, and 3 s curing protocols, respectively.

Considering the aforementioned variations in curing unit performance, strict standardization of radiant exposure was not of primary importance for kinetic investigations in the present study. As polymerization shrinkage stress development reached maximum rates shortly after the initiation of light-curing (within 1.7–4.1 s), the kinetic parameters R_DC_max and R_SS_max were less affected by variations in total radiant exposure, being primarily influenced by radiant exitance. Furthermore, the differences in radiant exposure were considered less important for the present study design as 1.5 mm thick specimens were expected to reach their plateau of maximum attainable conversion for all curing protocols [30]. The particular specimen thickness of 1.5 mm was chosen to obtain a ratio of bonded to unbonded surface area (“C-factor”) of 2.0 in order to evaluate the development of shrinkage stress under moderately constrained conditions [25]. Using the common clinical thickness of 4 mm for bulk-fill composites would increase the unbonded surface area, thus reducing the C-factor to only 0.75 and possibly underestimating shrinkage stress.

The idea that the same degree of conversion can be attained for the same radiant exposure, regardless of curing time and radiant exitance, termed “exposure reciprocity law”, has been held valid in the past when halogen curing units with low radiant exitances dominated dental practice. The reciprocity became disputed when high-intensity LED curing units were introduced, which led to departures from the reciprocal relationship [31]. These inconsistencies with the “reciprocity law” were explained by high-intensity light increasing the rate of bimolecular termination, reducing the number of polymer growth centers, and rendering the polymerization less efficient [32,33]. The susceptibility of a composite to being affected by this mechanism appears to depend on its viscosity, as for more viscous, i.e., more highly filled composites, bimolecular termination was shown to be less pronounced and exposure reciprocity held better than for flowable composites [34]. The higher sensitivity of flowable composites to a reduction of their degree of conversion by high-intensity light was also demonstrated in our study, as DC_5min_ values were more significantly affected by curing protocols for the flowable compared to the sculptable and the packable composites. Comparable deviations from exposure reciprocity in flowable composites were also observed in studies that compared the effect of radiant exitances of 1340 and 3440 mW/cm^2^ on degree of conversion [25] and microhardness [4].

Within a given material, the effect of curing protocols on DC_5min_ matched the differences observed in LS_5min_. If DC_5min_ values showed statistically significant differences among curing protocols, these differences were correspondingly reflected on LS_5min_ since the unconstrained shrinkage is directly dependent on the number of converted double bonds. The relationship between linear shrinkage and shrinkage stress is comparatively more complicated, as it results from simultaneously occurring volumetric changes accompanied by an increase in elastic modulus [35]. Our results indicate that the effect of curing protocols on DC_5min_ and LS_5min_ did not translate into statistically significant differences in SS_5min_ for any of the tested materials. The possible explanation is that due to the more complex nature of shrinkage stress development, the SS_5min_ data were more scattered than the data for DC_5min_ and LS_5min_, with respective coefficients of variability of 8.6–32.0%, 0.5–5.5%, and 1.3–7.1%. Relative magnitudes of these coefficients of variability (DC_5min_ < LS_5min_ < SS_5min_) reflect the relative complexities of material properties, with degree of conversion being the most fundamental property and thus the easiest to estimate experimentally. In contrast, the high scattering of shrinkage stress data resulted in less precise estimates and lower statistical power for identifying significant differences among curing protocols.

The results of inter-material comparisons for LS_5min_ reflect the fact that linear shrinkage is jointly determined by the resin ratio and degree of conversion. In addition to containing less resin available for volumetric change, more highly filled materials also exhibited lower DC_5min_ due to mobility limitations caused by higher viscosity; the interplay of these two factors resulted in LS_5min_ being reduced as the composite’s filler load increased. Hence, the most highly filled composites, SureFil and Filtek One (82 and 59 vol%), showed the lowest LS_5min_ among all tested materials, Tetric PowerFill (54 vol%) had intermediary LS_5min_ values, while the highest LS_5min_ was shown by the flowable composites Tetric PowerFlow, Filtek Supreme Flowable, and Tetric EvoFlow (46, 46, and 31 vol%, respectively). Additionally, LS_5min_ values were highly correlated with SS_5min_, which is the usually observed relationship for shrinkage evaluation in semi-rigid stress analyzers [36]. When constrained semi-rigidly, the flowable composites tend to develop higher shrinkage stress than the sculptable composites, as the shrinkage behavior under such conditions is more affected by linear shrinkage than elastic modulus [25]. In low-compliance stress analyzers, the opposite tends to be the case (sculptable composites show higher stress) because the shrinkage behavior is dominated by elastic modulus [37]. It can be argued that semi-rigid devices such as the stress analyzer used in our study can better simulate the clinical environment, owing to their capability to partially relieve shrinkage stress similar to the elasticity of cavity walls [27].

Kinetic parameters R_DC_max and R_SS_max were more discriminative among curing protocols than the static parameters obtained at the end of the 5 min observation period. The increase in radiant exitance from 1100 to 2850 mW/cm^2^ was accompanied by corresponding increases in R_DC_max (16.3–80.4%) and R_SS_max (24.8–73.1%). The highest increase in R_SS_max due to higher radiant exitances (73.1%) identified for the packable composite SureFil may be related to its very high filler load and consequently high elastic modulus [38]. However, despite the R_SS_max differences observed among the curing protocols for SureFil being the highest in relative terms, these differences appear practically less significant when considered in the context of R_SS_max values for other investigated materials, among which SureFil showed the lowest R_SS_max overall. As the other tested materials reached R_SS_max values more than twice that of SureFil, the wide range of stress values measured for other materials reduces the practical significance of otherwise pronounced relative differences observed within SureFil. Additionally, SureFil exhibited the lowest DC_5min_, LS_5min_, and SS_5min_ among all tested materials. The considerations mentioned in the example of SureFil are part of a more general observation that in a full factorial analysis the factor “material” was more influential compared to the factor “curing protocol”. Relative effect sizes of individual factors were quantified using partial eta-squared values, which confirmed material type as a more influential source of variance than differences in curing protocols. Hence, the choice of restorative material played a more important role in the development of potentially detrimental shrinkage forces on the tooth/restoration interface than variations in light-curing protocols. The higher practical significance of material type for shrinkage behavior is also apparent, with inter-material differences in static and kinetic shrinkage data being much more pronounced than the differences among curing protocols within the same material. It should also be mentioned that, similar to the findings from a previous study [25], the effect of curing protocols on the shrinkage behavior of the composites designed specifically for high-intensity light-curing (PowerFill and PowerFlow) did not differ from that observed for other composites.

In contrast to previous studies that measured shrinkage kinetics in real time over 15 min [25,26,27,29], the present study used a shorter observation period of 5 min, which was sufficient to evaluate the kinetic parameters of interest, as these were shown to be more affected by variations in curing conditions than final values measured at the end of the observation period. The results showed that considerable differences in kinetic parameters could be observed as a result of different curing protocols despite the comparatively modest effect of curing protocols on static parameters measured at the end of the observation period. Although the “static” SS_5min_ values were statistically similar within any given material regardless of the curing protocol, higher radiant exitances significantly accelerated shrinkage stress development, which may have negative effects on the adhesive interface integrity. As the investigated range of radiant exitances (1100–2850 mW/cm^2^) is becoming increasingly common in contemporary curing units [16], the related effects on shrinkage stress deserve further investigation. The tendency of dental equipment manufacturers to continuously increase the curing unit output has raised some concerns over the possible pulp damage due to temperature increase during light curing [39,40]; however, the available evidence indicates that high-intensity light-curing is tolerated by the pulp provided that the manufacturer’s directions are followed.

The fact that among all parameters of the double exponential sum function, the parameter “b” was affected the most by different radiant exitances indicates that faster initiation exerted the most pronounced effect on polymerization rate during the gel phase. For most materials, higher radiant exitance led to a lower extent of polymerization in the gel phase (parameter “a” decreased with higher radiant exitance), which was accompanied by a higher extent of polymerization in the glass phase (parameter “c” increased with higher radiant exitance). This general pattern was observed for all tested materials except for two materials specifically designed for high-intensity curing (PowerFill and PowerFlow), for which the relative extents of polymerization in the gel and glass phase were either unaffected by curing protocols or were affected in an opposite direction (i.e., gel phase showing a more extensive polymerization for higher radiant exitance for PowerFill). Additionally, most of the tested materials showed a consistent increase in the parameters “b” and “d” with higher radiant exitances, which evidences faster polymerization due to a higher initiation rate in both the gel and the glass phase, being aligned with the data for other kinetic parameters. However, PowerFlow again presented an exception, as its reaction rate parameters “b” and “d” were much less affected by polymerization protocols than any other material. The different behavior of PowerFill and PowerFlow may be related to their compositional modifications intended to make the materials suitable for high-intensity light-curing [8,9]. PowerFill features an AFCT reagent that modifies polymerization kinetics and affects the final structure of the polymer network. A compound of similar functionality is also included in Filtek One [10]; however, for this material no effect of AFCT-modified reaction on polymerization kinetics nor shrinkage stress development was identified in the present study. According to the manufacturer’s information, PowerFlow does not contain an AFCT-modifier, hence its specific kinetic behavior can be attributed to other compositional features of the resin system. The more pronounced influence of the material type in comparison to curing protocols mentioned for other variables was also observed for polymerization kinetic parameters of the double exponential sum function, again highlighting the fact that the selection of material had higher practical significance for polymerization and shrinkage kinetics than the parameters of curing protocols.

In a prequel study that used a similar methodology for evaluating linear shrinkage and shrinkage stress as the present study [25], the shrinkage behavior of the composites specifically designed for high-intensity light-curing was comparable to that of other composites from the same viscosity group. Similar observations were made in the present study; however, as the present study additionally evaluated polymerization kinetics, distinct behaviors of PowerFill and PowerFlow were identified through the evaluation of the double exponential sum function. This indicated that the composites specifically designed for high-intensity light-curing do present a different response to higher radiant exitances than the regular composites; however, these subtle differences were identifiable only in the real-time degree of conversion analysis and were not observed in the macroscopic evaluation of shrinkage stress.

## 5. Conclusions

This in vitro study showed that the polymerization shrinkage behavior of contemporary restorative composite materials of different viscosities cured with radiant exitances of up to 2850 mW/cm^2^ was overall more affected by material type than differences in curing conditions. In the practical sense, these results indicate that polymerization shrinkage stress can be more effectively modified by material selection than by variations in curing parameters. In addition to having lower practical significance compared to material type, different curing protocols showed a more pronounced effect on kinetic variables (polymerization rate and shrinkage stress rate) than static variables measured at the end of the observation period. Composites specifically designed for rapid (3 s) high-intensity light-curing differed from the other investigated materials with regard to polymerization kinetics while showing comparable behavior to other materials in terms of linear shrinkage and shrinkage stress.

## Figures and Tables

**Table 1:** Compositional details of investigated materials as provided by their respective manufacturers.

Composite Name	Filler Content (wt%/vol%)	Resin Matrix	Photoinitiator	Manufacturer	Manufacturer Recommended Curing Time	Shade/LOT No.
SureFil High Density Posterior Composite	82/66	Bis-GMA, UDMA, TEGDMA	CQ/amine	Dentsply Sirona, Konstanz, Germany	40 s with a minimum of 300 mW/cm^2^	A/00036175
Filtek One Bulk Fill	77/59	UDMA, aromatic UDMA, DDDMA, proprietary AFM	CQ/amine	3M, St. Paul, MN, USA	20 s for 550–1000 mW/cm^2^ 10 s for 1000–2000 mW/cm^2^	A2/NE12444
Tetric PowerFill	77/54	Bis-GMA, Bis-EMA, UDMA, propoxylated bisphenol A dimethacrylate, DCP	CQ/amine + Ivocerin + Lucirin TPO	Ivoclar Vivadent, Schaan, Liechtenstein	10 s for 900–1400 mW/cm^2^ 5 s for 1800–2200 mW/cm^2^ 3 s for 2700–3300 mW/cm^2^	IVA/Z01YP3
Tetric PowerFlow	68/46	Bis-GMA, Bis-EMA, UDMA	CQ/amine + Ivocerin	Ivoclar Vivadent, Schaan, Liechtenstein		IVA/YM0105
Filtek Supreme Flowable	65/46	Bis-GMA, TEGDMA, proprietary resin (“Procrylat”)	CQ/amine	3M, St. Paul, MN, USA	20 s for <1000 mW/cm^2^ 10 s for >1000 mW/cm^2^	A2/NC93129
Tetric EvoFlow	58/31	Bis-GMA, UDMA, decandioldimethacrylate	CQ/amine	Ivoclar Vivadent, Schaan, Liechtenstein	20 s for 500–800 mW/cm^2^ 10 s for >800 mW/cm^2^	A2/Z01M4P

**Table 2:** Statistical significance (*p*-values) and practical significance (partial eta-squared values) for the factors “material” and “curing protocol”.

Variable	Material		Curing Protocol		Material × Curing Protocol	
	*p*-Value	Partial η^2^	*p*-Value	Partial η^2^	*p*-Value	Partial η^2^
**Degree of conversion**	<0.001	0.973	<0.001	0.563	<0.001	0.436
**Linear shrinkage**	<0.001	0.991	<0.001	0.364	0.001	0.284
**Shrinkage stress**	<0.001	0.641	0.923	N/A	0.154	N/A
**Maximum polymerization rate**	<0.001	0.988	<0.001	0.889	0.007	0.274
**Maximum stress rate**	<0.001	0.820	<0.001	0.484	0.270	N/A
**Time of maximum polymerization rate**	<0.001	0.419	0.005	0.137	0.108	N/A
**Time of maximum stress rate**	<0.001	0.334	<0.001	0.451	0.096	N/A

## Data Availability

Not applicable.
